# The role of sex, age and genetic polymorphisms of CYP enzymes on the pharmacokinetics of anticholinergic drugs

**DOI:** 10.1002/prp2.775

**Published:** 2021-05-18

**Authors:** Shanna C. Trenaman, Susan K. Bowles, Melissa K. Andrew, Kerry Goralski

**Affiliations:** ^1^ Department of Medicine (Division of Geriatric Medicine) Nova Scotia Health Halifax Nova Scotia Canada; ^2^ Department of Medicine (Geriatrics) Dalhousie University Halifax Nova Scotia Canada; ^3^ Department of Pharmacy Nova Scotia Health Halifax Nova Scotia Canada; ^4^ College of Pharmacy Dalhousie University Halifax Nova Scotia Canada; ^5^ Department of Pharmacology Dalhousie University Halifax Nova Scotia Canada; ^6^ Department of Pediatrics Dalhousie University Halifax Nova Scotia Canada; ^7^ Division of Pediatric Hematology and Oncology IWK Health Centre Halifax Nova Scotia Canada

**Keywords:** aging, anticholinergics, pharmacokinetics, sex differences

## Abstract

There is evidence that use of drugs with anticholinergic properties increases the risk of cognitive impairment, and increased exposure to these drugs potentiates this risk. Anticholinergic drugs are commonly used even with associated risk of adverse events. Aging, sex, and genetic polymorphisms of cytochrome P450 (CYP) enzymes are associated with alterations in pharmacokinetic processes, which increase drug exposure and may further increase the risk of adverse drug events. Due to the increasing burden of cognitive impairment in our aging population and the future of personalized medicine, the objective of this review was to provide a critical clinical perspective on age, sex, and CYP genetic polymorphisms and their role in the metabolism and exposure to anticholinergic drugs. Age‐related changes that may increase anticholinergic drug exposure include pseudocapillarization of liver sinusoidal endothelial cells, an approximate 3.5% decline in CYP content for each decade of life, and a reduction in kidney function. Sex‐related differences that may be influenced by anticholinergic drug exposure include women having delayed gastric and colonic emptying, higher gastric pH, reduced catechol‐O‐methyl transferase activity, reduced glucuronidation, and reduced renal clearance and men having larger stomachs which may affect medication absorption. The overlay of poor metabolism phenotypes for CYP2D6 and CYP2C19 may further modify anticholinergic drug exposure in a significant proportion of the population. These factors help explain findings of clinical trials that show older adults and specifically older women achieve higher plasma concentrations of anticholinergic drugs and that poor metabolizers of CYP2D6 experience increased drug exposure. Despite this knowledge neither age, sex nor CYP phenotype are routinely considered when making decisions about the use or dosing of anticholinergic medications. Future study of anticholinergic medication needs to account for age, sex and CYP polymorphisms so that we may better approach personalized medicine for optimal outcomes and avoidance of medication‐related cognitive impairment.

## Graphical abstract

This review examines the effects of age, sex and genetic polymorphisms on the pharmacokinetics (absorption, distribution, metabolism, excretion) of anticholinergic medications. Increased drug exposure increases risk of adverse drug events; including cognitive impairment which is associated with anticholinergic medication use.

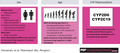

**Abbreviations**
5‐HMT: 5‐hydroxymethyl tolterodine
AUC: area under the curve
CYP: cytochrome P450
EM: extensive metabolizers
GPCR: G‐protein coupled receptor
IM: intermediate metabolizers
IV: intravenous
M: muscarinic
PM: poor metabolizers
UM: ultra‐rapid metabolizers

## 1 INTRODUCTION

Anticholinergic medications are potentially inappropriate for older adults.^1^, ^2^ Further to general prescribing guidelines^1^, ^2^ which caution against anticholinergic medication use in older adults, two academic groups (5th Canadian Consensus Conference on the Diagnosis and Treatment of Dementia^3^ and the Lancet Commission^4^) have recently identified anticholinergic medications among potential risk factors for developing dementia. Subsequent to the publication of these guidelines, several new studies have identified exposure to anticholinergic agents as risk factors for mild cognitive impairment and dementia.^5^, ^6^, ^7^ Clinical experience and research demonstrate an increased risk of adverse drug events,^8^, ^9^, ^10^, ^11^, ^12^ cognitive impairment,^13^, ^14^, ^15^, ^16^, ^17^, ^18^ and mortality^12^ related to the use of anticholinergic drugs in older adults. These adverse events can result in emergency department visits,^19^ hospital admission,^20^ or death^21^ with older adults being at increased risk of these sequelae.^22^, ^23^, ^24^ Due to variability in the anticholinergic activity of individual medications, one agent in isolation may fail to cause any noticeable effect but when two or three anticholinergic agents are combined the total anticholinergic burden can result in adverse events.^10^, ^17^, ^20^, ^21^, ^25^, ^26^, ^27^, ^28^ Total medication exposure or anticholinergic burden depends upon the pharmacokinetic factors in the subject relating to the particular medication(s) consumed.

Age, sex, and genetic variation in cytochrome P450 (CYP) enzymes^29^, ^30^, ^31^, ^32^, ^33^ are important factors leading to variability in drug metabolism and disposition. As a result, these factors may also contribute to variation in systemic drug exposure, response including resultant adverse events^22^, ^23^, ^24^ and toxicity to a variety of drugs including anticholinergic medications. This review supports clinical decision‐making surrounding anticholinergic medication use as we come to understand their potential risk for causing cognitive impairment and dementia, particularly in older female patients. Our analysis examines the effects of age, sex, and genetic polymorphisms of CYP2D6, CYP2C19, and CYP3A4 on the pharmacokinetics (absorption, distribution, metabolism, excretion), subsequent exposure, and pharmacologic response to anticholinergic medications. This review will provide clinicians with the pharmacokinetic considerations required when using anticholinergic medications.

## 2 METHODS

### 2.1 Data sources for review

**FIGURE 1:**
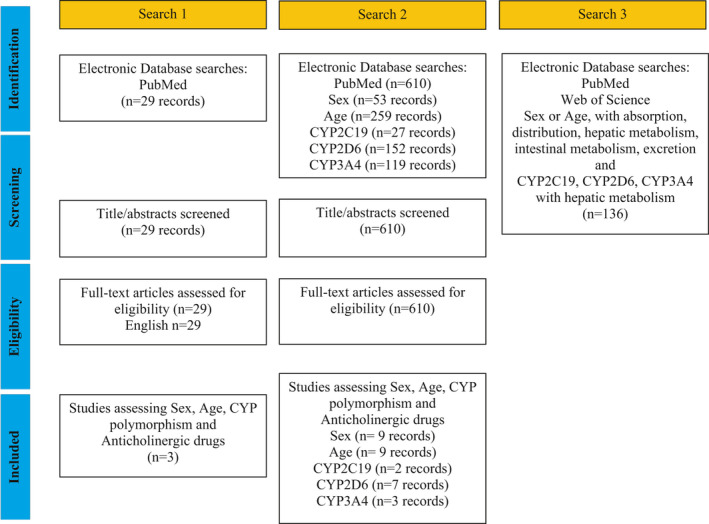
PRISMA style flow diagram

The PubMed database was searched across all available dates (1950–January 2020) with the initial search terms age, sex, anticholinergic agent, and pharmacokinetics. Anticholinergic agents were considered any drugs appearing on the anticholinergic cognitive burden scale^34^ as these were medications used commonly, and this anticholinergic scale is freely available for consultation. The preliminary search lacked recent studies including human subjects. A second search included limits of human subjects, English language, and clinical trials. In this directed search each term; sex, age, CYP2C19, CYP2D6, and CYP3A4 (the most common CYP enzymes involved in the metabolism of anticholinergic drugs) were searched in combination with anticholinergic and pharmacokinetics. Further searches were completed using the specific pharmacokinetic parameter of interest (absorption, distribution, metabolism, excretion) with each of the search terms sex, age, CYP2C19, CYP2D6, or CYP3A4. The Web of Science database was consulted to find citing articles. Further articles were taken from review articles examined during the literature searches. Details of the three searches are shown in Figure 1. This review was not meant to be an exhaustive summary of all available literature on the topic but instead a review of the literature to inform clinical decision‐making about anticholinergic drugs when used by older adults.

## 3 RESULTS

### 3.1 Anticholinergic receptors and signaling

The term anticholinergic agent refers to those drugs that antagonize the muscarinic acetylcholine (M) receptor. The M receptor is a G‐protein‐coupled receptor (GPCR) that resides on the cell membrane. It is comprised of seven alpha helices that span the cell membrane and an extracellular binding domain. When activated, the GPCR undergoes a conformational change that induces dissociation of the trimeric G protein‐complex into the free and active Gα and Gβγ subunits. The Gα and Gβγ subunits activate enzyme effectors or ion channels which regulate intracellular concentrations of secondary messengers such as cyclic adenosine monophosphate, guanosine 3′,5′‐cyclic monophosphate, diacylglycerol, inositol trisphosphate, diacylglycerol, arachidonic acid, sodium, potassium, or calcium depending on the receptor subtype.^35^ Gα and Gβγ activity is terminated by activation of an endogenous high‐affinity GTPase located in the Gα subunit, which hydrolyzes the terminal γ‐phosphate of Gα‐GTP to Gα‐GDP which then binds Gβγ to reform the trimeric G protein‐ complex.^36^, ^37^ In response to prolonged signaling, receptors can be internalized by separation from the effector and binding to small endosomes. This desensitizes the receptor by reducing the number of receptors on the cell surface. This occurs in response to receptor phosphorylation which is often related to a hormone response.^37^, ^38^ The five M receptor subtypes and their associated functional response to agonism and antagonism are described in Table 1. M1, M3, and M5 receptors all couple with Gq/11 and lead to release of calcium from the sarcoplasmic reticulum. M2 and M4 receptors are coupled to Gi proteins and their activation leads to inhibition of adenylyl cyclase.^39^, ^40^

**TABLE 1:** Description of the five muscarinic receptor subtypes, their distribution throughout the body, and effect of agonism or antagonism at each muscarinic receptor subtype

Receptor	Most common locations	Functional response (agonism)	Anticholinergic side effect (antagonism)
M1	Cerebral cortex, hippocampus and striatum, autonomic ganglia, gastric and salivary glands, enteric nerves	Increase cognitive function—learning and memory Increase seizure activity	Delirium, sedation, confusion
M2	CNS, heart, smooth muscle, autonomic nerve terminals	Heart—SA node: slowed spontaneous depolarization, hyperpolarization, decrease HR AV node: decrease conduction velocity Atrium: decrease refractory period, decrease contraction Ventricle: slight decrease in contraction	Increased heart rate, arrhythmia
M3	CNS, smooth muscle, and glands	Increase contraction (predominantly in bladder smooth muscle) Increase secretion (predominant in salivary glands) Increase tremor	Urinary retention, decreased salivation
M4	CNS forebrain	Inhibition of neurotransmitter release	Delirium, sedation, confusion
M5	Rare—CNS and periphery	Facilitates dopamine release Involved with drug seeking behavior	Reduced drug seeking

### 3.2 Serum anticholinergic activity and anticholinergic burden

M receptor antagonists have limited therapeutic use and are represented predominantly by bladder antispasmodics used to treat urinary incontinence. Many other medications have anticholinergic properties despite the M receptor not being the intended receptor for effect.^41^, ^42^ Such agents tend to have a lower level of anticholinergic activity. However, when multiple drugs with low levels of anticholinergic activity are combined the cumulative anticholinergic activity and anticholinergic burden increases.^8^, ^41^, ^43^, ^44^

Anticholinergic activity is dependent upon many factors, including the drug's binding to the M receptor, its absorption and distribution to tissues (including the brain), its concentration in circulation, intestinal and hepatic CYP metabolism and drug transport, the presence of any active metabolites that are produced, and the rate of elimination of the parent drug and active metabolites from the body. As pharmacokinetics can be affected by sex, age, or genetic polymorphisms (CYP enzymes), all these must be understood to quantify the total anticholinergic activity and rationalize the use of anticholinergic medications in clinical practice. Our findings are shared below and summarized in a table format in Appendix.

### 3.3 Sex

#### 3.3.1 Role of sex on the absorption of anticholinergic medications

Some, but not all, studies showed that gastric and colonic emptying was slowed in women, potentially increasing the oral bioavailability of some drugs.^45^, ^46^, ^47^, ^48^, ^49^, ^50^, ^51^ When stratified by age, the rate of gastric emptying for postmenopausal women and men was similar^52^ and significantly faster than premenopausal (younger) women.^50^ Gastric pH was higher in females^53^ which may increase absorption of basic medications such as tricyclic antidepressants, many of which are quite potently anticholinergic. This difference in gastric pH was quantified by Feldman and Barnett in 1991 as a mean pH of 2.79 for women and 2.16 for men, which was due to reduced acid secretion in women.^54^ The greater stomach size in men allowed for more fluid to be contained therein which may have improved both the rate and extent of dissolution of introduced oral dosage forms for men in comparison with women. By contrast, intestinal pH was not found to differ by sex.^55^, ^56^ CYP enzymes exist in intestinal enterocytes, where they contribute to the first‐pass metabolism of orally administered drugs. Intestinal CYP3A4 metabolism inconsistently exhibited sex differences. Early reports suggested that the CYP3A4 substrates verapamil and midazolam had increased bioavailability in women.^57^, ^58^, ^59^ However, in 2005 a detailed analysis of duodenal punch biopsies from 48 men and 45 women found no clinically meaningful sex difference in intestinal CYP3A4 content.^60^ Krecic‐Shepard et al. observed that oral verapamil was cleared more quickly in men with no significant difference after intravenous (IV) administration, suggesting some differences in intestinal metabolism exist^61^ which could affect those anticholinergic medications that are substrates of CYP3A4. In females, the CYP3A4 content in the intestine was shown to decrease by approximately 20% after menopause^60^ which may reduce CYP3A4 metabolism and affect the sex‐difference in CYP3A4 pharmacokinetics in older women. This decrease in intestinal CYP3A4 in postmenopausal women has not been shown to be clinically meaningful to date. Similarly, male versus female differences in the drug efflux pump ABCB1 (p‐glycoprotein) in the intestinal lumen was hypothesized as a contributor to differences in drug absorption between sexes,^60^ but this too has not been demonstrated to be clinically meaningful in studies to date.

#### 3.3.2 Role of sex on the distribution of anticholinergic medications

In general, males are larger than females across the lifespan, with increased height, body mass index, and waist circumference.^62^ Compared with men, women have increased adiposity. This difference in body composition has failed to show much difference in actual drug distribution and any differences attributable to body composition can largely be explained by differences in total body mass.^63^ Distribution of drugs to the brain was dependent upon the lipophilic nature of the blood‐brain barrier which favored passage and accumulation of lipophilic drugs. At this time, no statistically significant difference has been found between similarly aged women and men with respect to albumin permeability of the blood brain barrier^64^ which likely can be extrapolated to at least some medications. The brain is also protected by p‐glycoprotein, which prevents drugs from accumulating in the brain by pumping them from brain capillary endothelial cells to the blood.^65^ These mechanisms have not demonstrated any sex difference to date.

#### 3.3.3 Role of sex on the metabolism and transport of anticholinergic medications

Several studies supported that hepatic CYP metabolism varied between men and women although the clinical significance was a challenge to understand. The most abundant hepatic CYP enzyme, CYP3A4, was involved in the metabolism of some anticholinergic medications. In humans CYP3A4 had a higher level of protein expression in the female liver.^66^ Consistent with the expression data, CYP3A4 oxidation was reported to be more efficient in women^57^, ^67^ with a two‐fold higher CYP3A4 hepatic content and 50% increase in the metabolizing capacity^68^ but this finding has not been replicated in other scientific investigations.^69^, ^70^ An in vitro study from samples of 43 healthy livers in subjects between the ages of 27 and 83 showed a 24% increase in CYP3A4 activity identified by erythromycin N‐demethylation in females.^71^ Women had an increase in CYP3A4 activity measured as a greater clearance of CYP3A4 substrates such as the weakly anticholinergic antihypertensive medication nifedipine^72^ and the weakly anticholinergic sedative alprazolam.^73^ On average, the weight‐normalized clearance of alprazolam to active metabolites^74^ and nifedipine to inactive metabolites^75^ was mainly due to CYP3A4 and was 20%–30% higher in young women than in young men. This difference applied to both parenteral and oral administration and was not explained away by p‐glycoprotein activity.^76^ For context, CYP3A4 activity was studied in relation to metabolism of some non‐anticholinergic agents, such as midazolam to its active metabolites^77^ and clindamycin. Meta‐analysis suggested that women exhibited a 16% higher weight‐corrected oral clearance of midazolam (*p* < .001) and 20% higher systemic clearance (*p* = .002) than men. No significant difference in the area under the curve (AUC) after oral dosing of midazolam was found but after IV administration women showed lower AUC than men (*p* = .02). No sex‐dependent differences were observed in midazolam bioavailability.^78^ Clindamycin did not show any sex difference in its oral pharmacokinetics.^79^ The study of midazolam and clindamycin confirmed sex variability in CYP3A4 metabolism, but failed to demonstrate any consistent sex‐differences.

There was less study of sex differences in CYP2D6 and 2C19 metabolism identified in the literature search. Investigations of sex‐differences in CYP2C19 activity included 4‐hydroxymephenytoin, the active metabolite of the anticonvulsant mephenytoin, and zonisamide metabolism to its inactive metabolites^80^ which failed to show any sex‐differences.^81^, ^82^ A Spanish study examining caffeine metabolism found higher CYP2D6 activity in women.^83^

Sex differences were demonstrated in the glucuronidation of some medications (acetaminophen) but not others (zidovudine),^84^, ^85^, ^86^ suggesting that sex differences in drug conjugation exist and are drug‐dependent. To date, no anticholinergic agents have been explored with respect to glucuronidation. Clearance of some non‐anticholinergic drugs by glucuronidation were shown to be increased in men in comparison with women including oxazepam,^67^ temazepam,^87^ and acetaminophen.^88^ With regard to catechol‐O‐methyltransferase activity, liver tissue from female subjects exhibited approximately 25% lower activity than samples from male subjects.^89^ There was a two‐fold greater expression of hepatic p‐glycoprotein in men compared with women^90^ with unclear clinical relevance.

#### 3.3.4 Role of sex on the renal elimination of anticholinergic medications

Glomerular filtration is related to body mass. Males typically have a greater body weight than females,^62^ so generally glomerular filtration is greater in males than females. This likely explains most sex‐differences in renal drug clearance, though this was not observed for all drugs. Sex was found to be a significant factor in methotrexate clearance, with a 17% reduction in females after standardizing doses for body weight.^91^ Some authors reasoned that for narrow therapeutic index drugs, the sex‐related effect on kidney function may be clinically relevant.^91^, ^92^ Pharmacokinetic studies confirmed sex‐differences in renal clearance for many drugs including the weakly anticholinergic drug digoxin, which had slower clearance in females^93^ and the moderately anticholinergic drug amantadine, which had been shown to have significantly higher renal clearance in men due to putative sex differences in renal tubule secretion by organic cation transporters.^94^

Sex differences in pharmacokinetics have been explored with respect to some anticholinergic medications. Results of studies that examined sex differences in anticholinergic drug pharmacokinetics as their primary objective are listed in Table 2.

**TABLE 2:** Details of study population, study objectives, methodology, and results of trials identified to have a primary objective of exploring sex‐differences in pharmacokinetic parameters for anticholinergic medications

Study author & design	Study population	Study objective	Methodology	Results
Vicente et al.^97^ Randomized single‐lind controlled trial	24 healthy non‐smoking volunteers (12 women and 12 men), 18–35 years old	To determine if quinidine induced prolongation of the time from the peak to the end of the T‐wave is greater in women than men	Subjects received either 4 mg/kg of quinidine IV or a matching placebo solution over 20 min with 28 blood samples and simultaneous ECGs collected after drug/placebo infusion for each subject at predetermined time points over the following 12 h	Quinidine causes QTc prolongation and T‐wave morphology changes in both women and men Quinidine‐induced maximum QTc (541 ± 40 ms vs. 510 ± 38 ms; *p* = .07) or maximum *T*_peak_–*T*_end_ (216 ± 60 ms vs. 222 ± 37 ms; *p* = .76) was similar for men and women There was a trend toward a lower maximum serum quinidine concentration in women compared with men (2.9 ± 0.7 μg/ml vs. 3.7 ± 1.2 μg/ml; *p* = .07) The slope describing serum quinidine concentration versus QTc prolongation was greater in women than in men (38 ± 10 ms/μg/ml vs. 28 ± 9 ms/μg/ml; *p* = .02) Differences between women and men occurred primarily in the first 20 min after quinidine infusion, when serum quinidine concentrations were higher in men than women
Benton et al.^95^ Randomized single‐blinded controlled trial	24 healthy non‐smoking volunteers (12 women and 12 men), 18–35 years old	To determine if women have larger increases in QT interval than men at equivalent serum concentrations of quinidine after intravenous administration	Subjects received either 4 mg/kg of quinidine IV or a matching placebo solution over 20 min. 28 blood samples and simultaneous ECGs were collected after drug/placebo infusion for each subject at predetermined time points over the following 48 h	There was a trend to greater weight‐adjusted clearance of quinidine in women than in men (5.2 ± 1.1 ml/min/kg vs. 4.3 ± 1.6 ml/min/kg) There was also a trend to a higher maximal plasma concentration of quinidine in men than in women (3.67 ± 0.13 μg/ml vs. 2.78 ± 0.87 μg/ml; *p* = .07) There were no sex‐related differences in the ratio of the AUC_∞_ of 3‐hydroxyquinidine to the AUC_∞_ of quinidine The estimated volume of distribution (*V*_d_) at steady state was not different between the men and women There was no difference in the free fraction of quinidine in serum between men and women The free fraction of 3‐hydroxyquinidine was slightly higher in women than in men (0.53 ± 0.05 μg/ml vs. 0.47 ± 0.05 μg/ml; *p* < .01)
Winchell et al.^98^ A series of open‐label, three‐period, randomized, crossover studies	1. 24 healthy young subjects (mean age: 25.5 years; range: 19–39 years; 16 males and 8 females 2. 18 healthy subjects (mean age: 28.7 years; range: 22–40 years; 8 males, 10 females) 3. 12 elderly subjects (mean age: 71.3 years; range: 65–79 years; 6 males, 6 females	To investigate the pharmacokinetics and bioavailability of cyclobenzaprine, including the effects of sex and age	1. Bioavailability: Subjects received 5 mg orally or 1.25 mg IV cyclobenzaprine 2. Pharmacokinetics: Subjects received a single oral dose of 2.5, 5, or 10 mg cyclobenzaprine on Day 1 then every 8 h from Days 8 through 14 with final dose on Day 15 3. Pharmacokinetics in aging: Subjects received 5 mg cyclobenzaprine orally three times daily for 7 days and a final dose on Day 8	1. Plasma concentrations increased initially, peaking at 4 h post dose, and then declined slowly Mean plasma clearance was 689 ± 216 ml/min—Mean oral bioavailability 5 mg tablet formulations were 0.55 (90% CI [0.51, 0.60]) 2. There were no statistically significant differences between males and females for any of the pharmacokinetic parameters—AUC_(0–8 h)_ and *C*_Max_ after the last dose were marginally significantly different between sexes 3. The population‐by‐sex effect was marginally significant for AUC_(0–8 h)_ (*p* = .056) but not for *C*_Max_
El‐Eraky et al.^96^ Open trial	48 healthy volunteers (27 men, 21 women) aged 18–64 years	To determine why women are more susceptible to QT interval prolongation and torsade de pointes after administration of drugs that delay cardiac repolarization	All subjects took quinidine sulfate capsules 3 mg/kg orally then ECGs and blood samples for quinidine concentrations were taken over 24 h following drug administration	There were no significant differences in quinidine concentrations between men and women or in any of the pharmacokinetic variables measured The QT_a_, and QT_c_ intervals were larger in females than in males Quinidine did not affect QRS duration in women but reduced QRS duration in men
Koren et al.^122^ Single‐center, single dose open‐label, reference replicate bioavailability study	12 healthy males and 12 healthy females, 18–45 years with a body mass index between 19–30 kg/m^2^	To determine the effect of sex on the pharmacokinetics of doxylamine–pyridoxine 10–10 mg delayed‐release tablets	Participants were given doxylamine–pyridoxine 20–20 mg delayed‐release tablets with 240 ml water on an empty stomach with blood sampling starting 1 h pre‐dose with samples analyzed using high performance liquid chromatography‐ tandem mass spectrometry	Females had significantly larger AUC_0–_ *_t_* for doxylamine compared with males A higher *C*_Max_ for doxylamine was observed in females compared with males
Malhotra et al.^118^ Two randomized double‐blind placebo‐controlled trials	1. 32 healthy males aged 18–45 years 2. 16 young men, 16 older men and 16 older women	To examine the effect of age, sex and race on the pharmacokinetics, pharmaco‐dynamics and safety profiles of fesoterodine	Subjects received either 8 mg of fesoterodine extended release or placebo with blood samples drawn over 36 h after drug administration and saliva samples on cotton wool collected over 24 h after drug administration	No apparent differences in *C*_Max_, AUC_0–∞_, *t*_max_, or mean residual time between males and females Total plasma clearance was highest in young men and lowest in older women Elderly women experienced a 1 g decrease in salivary volume and elderly men did not 5 h after dose Elderly men experienced the greatest residual urinary volume increase 8 h after dose
Ebert et al.^123^ Open label crossover study	7 men and 7 women of mean age 23 years and in good health	To identify any pharmacokinetic differences between male and female volunteers in the metabolism of scopolamine when given with grapefruit juice	Each subject received at random scopolamine 0.5 mg IV, scopolamine 0.5 mg orally, or scopolamine 0.5 mg orally mixed with 150 ml fresh grapefruit juice and blood sampling occurred over the 24 h following drug administration	*C*_Max_ was significantly higher in males than females (6.61 ng/ml vs. 3.93 ng/ml) after IV infusion All other parameters were similar
Macleod et al.^99^ Open label study	4 men and 5 women aged 21–30 years, and 5 older men and 5 older women aged 70–88 years	To identify age and gender differences in diazepam pharmacokinetics	10 ml blood samples were taken over 1 week after receiving 0.125 mg/kg diazepam IV over 10 min	There was a significant difference in plasma clearance between men and women (male: 33.2 ml/min and women: 18.1 ml/min) The half‐life in men (32 h) was significantly shorter than in women (46.2 h) *V*_d_ was not significantly different between sexes
Bigos et al.^100^ Naturalized prospective study	332 men and 191 women who were using olanzapine for AD or schizophrenia	To evaluate population pharmacokinetics of olanzapine and factors that contribute to variability in exposure including sex, race and smoking status	Plasma levels of olanzapine were determined and then used to calculate non‐linear mixed effects modelling for pharmacokinetic analysis	Men cleared olanzapine 38% faster than women (*p* < .0001, unpaired *t* test)
Hartter et al.^105^ Prospective study	15 male and female participants with major depression	To assess sex differences in fluvoxamine serum concentration at two different fixed dosing regimens (50 twice daily and 100 mg twice daily)	Drug monitoring after 14 days of either treatment	There was a significantly greater increase in fluvoxamine serum concentration in men than in women when the dose doubled (4.6‐fold vs. 2.4‐fold increase)

Abbreviations: AUC, area under the curve; IV, intravenous.

#### 3.3.5 Summary of studies showing sex‐differences in pharmacokinetics: Quinidine

The most commonly reported anticholinergic medication with a focus on sex‐related differences was quinidine, exploring drug‐induced QT interval prolongation (Table 2).^95^, ^96^, ^97^ The findings of both Benton and Vicente^95^, ^97^ suggested that women cleared quinidine at a faster rate than men. Unexpectedly, women had a more rapid onset of ECG changes in response to drug activity than men, which was not entirely explained by increased quinidine clearance. These studies demonstrated sex‐differences in quinidine pharmacokinetics; however, the mechanism of this difference was not clear.^95^, ^96^, ^97^ It was possible hormonal influences or rapid distribution after IV infusion contributed to the faster onset of activity in women which normalized over time to reach equilibrium between the sexes.

#### 3.3.6 Summary of studies showing sex‐differences in pharmacokinetics: Psychoactive medications

Many anticholinergic psychoactive medications were investigated for sex‐differences in absorption, distribution, metabolism, and excretion. A study of cyclobenzaprine examined sex‐differences using a series of open‐label, three‐period, randomized, crossover studies. The first study included 24 healthy young subjects (mean age: 25.5 years), the second 18 healthy subjects (mean age: 28.7 years), and the third 12 older subjects (mean age: 71.3 years). The primary objective was to investigate the bioavailability and pharmacokinetics of cyclobenzaprine with attention to the effects of sex, age, and hepatic insufficiency (Table 2). There were small significant differences in the AUC and *C*_Max_ for cyclobenzaprine between sexes in the older group.^98^ This is most likely due to accumulation of drug in the group of older females. A study of the benzodiazepine diazepam demonstrated a shorter *t*_1/2_ and a greater plasma clearance in men in comparison with women (Table 2).^99^ In a population of men and women receiving olanzapine for Alzheimer's disease or schizophrenia, between one and six samples were analyzed from each individual to determine sex‐differences in olanzapine clearance. Sex was found to be responsible for 12% of variability in olanzapine elimination. Men cleared olanzapine 38% faster than women.^100^ A natural pharmacokinetic study of anticholinergic antidepressants in older adults looked for sex‐differences in serum concentrations. The ratio of absolute serum concentration in comparison with the dose‐adjusted serum concentration was 1.1‐ to 1.5‐fold higher in women than in men for clomipramine and trimipramine. This was despite a dose reduction in females who received 10%–30% lower dose but still achieved serum levels equivalent to male participants.^101^ Findings of Mundo and Unterecker et al. suggested that clomipramine levels were not related to sex,^102^, ^103^ but rather the metabolites of clomipramine accumulated contributing to the higher plasma levels seen in women. A second naturalistic study of antidepressants that examined 19,870 blood samples failed to show a difference for the tricyclic antidepressants clomipramine or fluvoxamine^104^ which is in keeping with findings of Mundo and Unterecker.^102^, ^103^ However, in a study that examined dose regimens of fluvoxamine separately, a dose‐dependent sex difference in serum fluvoxamine concentration was observed. At a 100 mg daily oral dose, women achieved higher serum fluvoxamine concentrations than men, but with a 200 mg daily oral dose the serum concentrations were no longer statistically significantly different.^105^ This may relate to a saturable metabolizing enzyme that was in a greater concentration or more active in men. Sex was correlated to paroxetine plasma concentrations in three studies that examined the effect of sex on paroxetine pharmacokinetics. In a study of 171 subjects aged ≥70 years, men had a higher paroxetine *V*_d_ (461 ± 260 L) compared with women (346 ± 256 L).^106^ In a study of 1677 older men and women, the serum concentration of paroxetine was 32% higher in women (86 nmol/L vs. 65 nmol/L, *p* < .001).^104^ In a third study of 70 patients, the plasma concentration of paroxetine was higher in women across age groups (28 ng/ml vs. 16 ng/ml; *p* = .001).^107^ The mean AUC and *C*_Max_ for bupropion, a mildly anticholinergic antidepressant, were higher in women than men; however, once these parameters were standardized for body weight the statistical significance was lost.^108^ For bupropion, older women had a larger *V*_d_ and longer *t*_1/2_ than young men. This does make it challenging to know how much of the effect was attributable to sex versus age.^109^ Amitriptyline plasma levels were higher in women in a study of 110 inpatients receiving routine doses of amitriptyline,^110^ but no significant sex‐difference in serum concentration of amitriptyline was noted in the study by Reis et al.^104^ Nortriptyline plasma levels were affected by sex with females experiencing higher plasma levels.^111^ Desipramine was shown to have a longer elimination *t*_1/2_ and a faster oral clearance in older men than in older women.^112^ When examining risperidone plasma concentrations, the only parameter to exhibit a statistically significant difference between males and females was the plasma concentration/dose ratio. When weight was used to adjust the plasma concentration, any difference was lost.^113^ Many of these psychoactive medications are metabolized by CYP2D6, and a sex‐related difference in CYP2D6 activity has not consistently been identified in the literature,^114^ which means there are likely other sex‐dependent mechanisms contributing to these pharmacokinetics differences. In summary, while many sex‐differences exist in the pharmacokinetics of psychoactive anticholinergic medications, no consistent patterns were identified. The small increases in drug exposure that were identified (most often by women) may help explain the increased experience of adverse events by women.^115^, ^116^

#### 3.3.7 Summary of studies showing sex‐differences in pharmacokinetics: Bladder anticholinergics

Oxybutynin, the prototype bladder anticholinergic, is metabolized by CYP3A4 to N‐desmethyloxybutynin. This metabolite of oxybutynin is considered to cause many of the adverse events related to oxybutynin treatment, so understanding any role of sex in the metabolism of oxybutynin is important. Increased CYP3A4 activity and slowed renal elimination in women may increase exposure to the metabolite and increase the likelihood of adverse drug effects. However, an older study of oxybutynin pharmacokinetics failed to show any sex differences in the pharmacokinetics of oxybutynin or its active metabolite.^117^

Two randomized double‐blind placebo‐controlled trials assessed the effects of age, sex, and race on the pharmacokinetics and safety profiles of fesoterodine in 32 healthy males aged 18–45 years (16 white and 16 black men) and 16 young men, 16 older men and 16 older women (Table 2). Total plasma clearance of fesoterodine was highest in young men and lowest in older women, but there were no apparent sex differences in *C*_Max_, AUC_0–∞_, or *t*_Max_. Interestingly, 5 h after the dose was given, older women experienced a 1 g decrease in salivary volume whereas older men did not, which provided some evidence that women were more likely to experience adverse effects (e.g. dry mouth) from this anticholinergic medication use. There was no clinically meaningful difference in any of the pharmacokinetic parameters studied based on race (mean AUC_0–tz_ was 70.7 ng/ml × h in white and 64.1 ng/ml × h in black men, and mean *C*_Max_ was 6.1 ng/ml in white and 5.5 ng/ml in black men).^118^ Similarly, in a study of 337 individuals, darifenacin clearance was about 30% lower in females.^119^ No sex differences in pharmacokinetics had been identified for solifenacin^120^ or tolterodine.^118^ Trospium demonstrated an unexplained prolonged *t*_1/2_ in women compared with men.^121^ This collection of studies demonstrates the complex influence of sex on pharmacokinetics of bladder anticholinergics which are frequently used by older adults.

#### 3.3.8 Summary of studies showing sex‐differences in pharmacokinetics: Antihistamines

A single‐center, single‐dose, open‐label, reference replicate, bioavailability study in 12 healthy males and 12 healthy females aged 18–45 years with a body mass index between 19 and 30 kg/m^2^ was completed to determine the effect of sex on the pharmacokinetics of doxylamine–pyridoxine 10–10 mg delayed‐release tablets. Females had significantly larger AUC_0–_ *_t_* and a higher *C*_Max_, for doxylamine compared with males.^122^

#### 3.3.9 Summary of studies showing sex‐differences in pharmacokinetics: Scopolamine

An open‐label crossover study of seven men and seven women of mean age 23 years and in good health was completed to identify any sex differences in pharmacokinetics in the metabolism of 0.5 mg scopolamine when given IV or orally with or without grapefruit juice. The *C*_Max_ was significantly higher in males than females (6.61 ng/ml vs. 3.93 ng/ml) after IV infusion with all other parameters being similar.^123^ No sex differences were found in urinary elimination of scopolamine for any of the three different routes of administration.

### 3.4 Age

#### 3.4.1 Role of age on the absorption of anticholinergic medications

Gastric and colonic transit was significantly faster in postmenopausal women in comparison with premenopausal women,^50^ which suggested altered absorption. In a study of 16 healthy adults average age 81 years and 16 healthy adults average age 24 years, advanced age did not influence gastric emptying or small intestinal transit but older individuals had a slower colonic transit.^46^

#### 3.4.2 Role of age on the metabolism and transport of anticholinergic medications

In humans, it was well established that total hepatic CYP enzyme levels decline from about age 40 onwards. This had been quantified as about a 3.5% decline in CYP enzyme content for each decade of life potentially influencing the elimination of anticholinergic drugs undergoing metabolism by the CYP enzyme system, resulting in greater exposure to these agents.^69^, ^124^ An older study investigating the metabolic ability of CYP enzymes across a variety of ages revealed that CYP3A4 activity was reduced in older adults. The microsomal content of CYP3A4 was found to decrease by approximately 8% per decade of life.^69^ This trial failed to show a difference in CYP1A2, or CYP2C based on age. An in vitro study of healthy human liver samples obtained during surgical procedures from 43 subjects between the ages of 27 and 83 showed no variation in CYP3A4 activity in relation to age. In this study, CYP3A4 activity was quantified by measuring erythromycin N‐demethylation. While erythromycin N‐demethylation had been shown to decline with age, the results of this study suggested that the age‐related decline in enzyme activity was not due to declining CYP3A4 activity. Rather, other patient factors such as renal blood flow, renal filtration, or body composition were likely contributing.^71^ In females, intestinal CYP3A4 content had been shown to decrease by approximately 20% after menopause,^60^ which may have reduced intestinal CYP3A4 metabolism and contributed to an age‐dependent difference in CYP3A4 metabolism. Possibly due to a lack of studies, this decrease in intestinal CYP3A4 in postmenopausal women has not been shown to be clinically meaningful to date. Decreases in the clearance of CYP3A4 substrate drugs suggested that older people may experience increased adverse effects due to reduction in clearance of drugs that rely on CYP3A4 for metabolism prior to elimination.^125^

Drug conjugation was shown in several studies to remain fairly constant with respect to age.^126^ Undeniably, numerous factors such as genetics, medication use, and frailty^127^, ^128^ can influence glucuronidation and sulfonation, but in younger and older healthy people glucuronidation and sulfonation were not statistically significantly different. In aging rat models, liver sinusoidal endothelial cells undergo pseudocapillarization,^129^, ^130^ a process characterized by loss of sinusoidal fenestrations, thickening of the endothelium, perisinusoidal collagen deposition, and basal lamina formation.^131^ This process suggested that drug passages through the liver were reduced in size which, in theory, could prevent large molecules, in particular protein therapeutics and extensively protein‐bound drugs, from travelling through the liver and being cleared; this was shown for liposomal doxorubicin in aged rats compared to young rats.^132^ The relevance of these changes to anticholinergic drug pharmacokinetics in humans remains to be determined.

#### 3.4.3 Role of age on the renal elimination of anticholinergic medications

Renal elimination declines with age by all renal routes (glomerular filtration, tubular secretion, and passive reabsorption).^133^, ^134^ Any anticholinergic agent that is renally eliminated or has renally eliminated active metabolites is likely to accumulate in older adults in comparison to younger adults.

#### 3.4.4 Role of age on blood–brain barrier function

In men the *V*_d_ of (R)‐[11C] verapamil, a known p‐glycoprotein substrate, increased with age in several cortical brain regions, strongly suggesting a progressive decrease in blood brain–barrier p‐glycoprotein function with age.^135^ This could affect drug introduction to the brain which may affect efficacy or toxicity depending upon the anticholinergic agent used.

Studies with a primary objective of identifying age‐related differences in drug pharmacokinetics were listed in Table 3.

**TABLE 3:** Details of study population, study objectives, methodology, and results of trials identified to have a primary objective of exploring age‐related differences in pharmacokinetic parameters for anticholinergic medications

Study author & study design	Study population	Study objective	Methodology	Results
Winchell et al.^98^ A series of open‐label, three‐period, randomized, crossover studies	1. 24 healthy young subjects (mean age: 25.5 years; range: 19–39 years; 16 males and 8 females) 2. 18 healthy subjects (mean age: 28.7 years; range: 22–40 years; 8 males, 10 females) 3. 12 older subjects (mean age: 71.3 years; range: 65–79 years; 6 males, 6 females)	To investigate the pharmacokinetics and bioavailability of cyclobenzaprine, including the effects of age and hepatic insufficiency	1. Subjects received 5 mg orally or 1.25 mg IV cyclobenzaprine 2. Subjects received a single oral dose of 2.5, 5, or 10 mg cyclobenzaprine on Day 1 then every 8 h from Days 8 through 14 and a final dose on Day 15 3. Subjects received 5 mg cyclobenzaprine orally three times daily for 7 days and a final dose on the 8th day	Cyclobenzaprine plasma concentrations after multiple dosing were significantly higher for the older compared with young subjects After the first dose, plasma concentration profiles were similar in older and young subjects Mean accumulation ratio was 7.9 for older subjects compared with 4.3 for young subjects, and mean effective *t*_1/2_ was 33.4 h (range: 20.0–53.4 h) in older subjects compared with 18.4 h (range: 9.3–41.3 h) in young subjects
Malhotra et al.^118^ Two randomized double‐blind placebo‐controlled trials	1. 32 healthy males aged 18–45 years 2. 16 young men, 16 older men and 16 older women	To examine the effect of age, sex, and race on the pharmacokinetics, pharmacodynamics and safety profiles of fesoterodine	Subjects received either 8 mg of fesoterodine extended release or matching placebo with blood samples drawn over 36 h after drug administration	Renal clearance was 28% lower in older men and women than younger men

#### 3.4.5 Summary of studies showing age‐differences in pharmacokinetics: Psychoactive medications

Risperidone and its 9‐hydroxyrisperidone metabolite are active and have anticholinergic properties. In a study of 129 adults on risperidone maintenance therapy grouped by age (<45, 45–60, and >60 years), the risperidone maintenance dose was lowest in the oldest age group, but the unadjusted plasma risperidone concentrations did not differ significantly across age groups. However, when adjusted for subject body weight or maintenance dose, the plasma risperidone concentration was significantly higher in the older group. The concentration of active drug comprised both the 9‐hydroxyrisperidone metabolite and risperidone parent drug, with the difference driven by the 9‐hydroxyrisperidone concentration.^136^ This supported the use of the lowest dose possible of risperidone in older adults and provided support for the “start low and go slow” approach to antipsychotic dosing in geriatric populations. In comparison, the clearance of the sedative diazepam was not found to be affected by age in a study of young (21–30 years) males and females in comparison to older males and females (70–88 years).^99^ A naturalized study of multiple anticholinergic antidepressants showed an increase in the absolute serum concentrations to dose‐adjusted serum concentrations for fluvoxamine (2‐fold), amitriptyline, and clomipramine (1.5‐fold) in the oldest age group (those more than 65 years of age) in comparison to controls <40 years. No significant age difference was observed for the dose‐adjusted fluoxetine and trimipramine serum concentrations. For fluoxetine and trimipramine users, older adults were using 10%–30% lower total daily doses. The concentration to dose ratio of nortriptyline was two‐fold higher in adults over 65 in comparison with the controls <40 years old^101^; clearance was correlated with age with faster clearance at younger ages. No significant difference was found between patients younger than or older than 60 years in the mean dose‐corrected serum concentration of clomipramine and N‐clomipramine, which contradicted the findings of Waade.^103^ However amitriptyline plasma levels were higher in older adults than younger subjects,^110^ which was consistent with findings of Waade et al. and Dawling et al. who showed that both amitriptyline and nortriptyline levels were higher in older adults, with older women experiencing a more exaggerated effect than their male comparators.^137^ At daily oral doses of 100 or 200 mg, fluvoxamine serum concentration did not correlate with age.^105^ There was a trend to higher serum concentrations in older female patients with the lower dosage of fluvoxamine, but this diminished when the dosage was doubled and suggested there was an interaction between age and sex on fluvoxamine pharmacokinetics. Older subjects taking oral paroxetine had higher plasma concentrations than younger subjects.^138^ In a study that examined bupropion kinetics in older adults with depression (mean age 71.5 years), clearance was 80% of that seen in younger adults^109^ and *t*_1/2_ was 34 h in comparison to most sources which report 11–14 h.^109^, ^139^ Among females, there was no significant difference between young and older groups in any of the pharmacokinetic variables for triazolam. Among males, the *t*_1/2_ of triazolam increased. Furthermore, when age was evaluated as a continuous variable, AUC for triazolam increased significantly with age (*p* = .02) and clearance decreased with age (*p* = .02). Further examination of cyclobenzaprine pharmacokinetics showed increased *t*_1/2_ in older versus younger adults.^98^

#### 3.4.6 Summary of studies showing age‐differences in pharmacokinetics: Bladder anticholinergics

The potently anticholinergic drug oxybutynin followed the trend of increasing peak plasma levels and bioavailability with increasing age and frailty.^140^ This effect was so significant that study authors suggested halving the dose of oxybutynin for older adults to achieve the same plasma levels as younger adults. AUC and *C*_Max_ were increased 20% and 16% respectively when an older population was given the same dose of oxybutynin as a younger population. Moreover, solifenacin, a newer bladder anticholinergic, had a longer *t*_1/2_ due to slower elimination and longer time to reach *C*_Max_ in older adults. This could be explained by the slowed absorption of solifenacin in older adults which increased their exposure to solifenacin by about 1.2‐fold.^120^ In a study of 16 young men, 16 older men and 16 older women, receiving either 8 mg of fesoterodine extended release or matching placebo, the renal clearance of fesoterodine was 28% lower in older men and women than younger men^118^ (Table 3). This increased exposure to fesoterodine in older adults may predict increased exposure of tolterodine in older adults as well, as fesoterodine and tolterodine are related compounds, with both being metabolized to the same active ingredient.

#### 3.4.7 Summary of studies showing age‐differences in pharmacokinetics: Scopolamine

Healthy adult subjects were given scopolamine hydrobromide 0.5 mg IV if they were under 65 years of age and 0.3 mg if older than 65 years. These subjects then received a battery of tests of cognitive function in addition to measurement of pharmacokinetic variables. Older age was associated with slowed clearance and increased exposure to scopolamine. Age‐related increases in scopolamine exposure was likely the greatest contributor to the increased sensitivity to cognitive adverse effects in older adults. The study authors hypothesized that age‐related changes in CYP3A4 activity or content may have been responsible for the increased scopolamine exposure in older adults.^141^

### 3.5 Genetics

In addition to age and sex, it is important that we understand how genetic variation in CYP activity could influence clinical effect or toxicity as drugs that are substrates for these enzymes are frequently used by older adults.^29^

#### 3.5.1 CYP2D6

Genetic variation in the *CYP2D6* gene has been well characterized and identified 120 *CYP2D6* variants (alleles) that have altered levels of CYP2D6 enzyme activity. These alleles result from point mutations, deletions or additions, gene rearrangements, and deletion or duplication/multiplication of the entire gene and have different distributions among various ethnic groups. Phenotypically, individuals with two normal CYP2D6 alleles are extensive metabolizers (EMs), those with one normal and one poor metabolism allele are intermediate metabolizers (IMs) and those with two reduced metabolism alleles are poor metabolizers (PMs). For CYP2D6, there is a fourth phenotype, the ultra‐rapid metabolizers (UMs) which have at least one active CYP2D6 gene duplication. Of interest, PM variants are common in East Asian populations and exist across the world. Understanding the effect of these CYP2D6 variants on pharmacokinetics is important for predicting drug effect and adverse effect.

The effect of CYP2D6 phenotype on anticholinergic medication exposure had been investigated in older adults. CYP2D6 phenotypes had been well characterized with respect to codeine pharmacokinetics. Limited activation and effect of codeine occurred in CYP2D6 PMs, and increased metabolism and toxicity was reported in UMs.^142^ Nortriptyline plasma levels were mostly correlated to CYP2D6 genotype and sex.^111^ In nursing home patients exposed to anticholinergic drugs, the highest serum anticholinergic activity was found in groups of CYP2D6 PMs.^143^ Analysis of risperidone metabolism in 70 healthy volunteers (of whom 82.9% were either IM or EM) revealed that polymorphisms of the CYP2D6 enzyme were much more responsible than sex for variation in risperidone metabolism. CYP2D6 phenotype explained 52% of interindividual variability in risperidone pharmacokinetics. The AUC of the active moiety was found to be 28% higher in CYP2D6 PM compared with IM, EM, and UM. No other genetic markers were found to significantly affect risperidone concentrations.^144^ This genetic variation in the metabolism of risperidone was of such magnitude that it could alter results when conducting bioequivalence studies.^145^ Differences in dose responses should be considered as clinically relevant for any person initiated on risperidone, further supporting using the lowest possible doses at all times.

The bladder anticholinergic tolterodine is metabolized to a similarly active 5‐hydroxymethyl tolterodine (5‐HMT) by CYP2D6. The bioavailability of tolterodine was strictly related to the genetic polymorphism of CYP2D6 and it ranged from 10% to 74%.^146^ Byeon et al. investigated the relationship between CYP2D6 phenotypes and tolterodine pharmacokinetics in 46 Korean subjects. The single dose and multiple dose *C*_Max_ and AUC_0–24_ of tolterodine, respectively, were significantly higher in the PM groups than in the EMs. The ratio of clearance to bioavailability of tolterodine in the EMs was 5‐ to 18‐fold higher than PM (variant dependent) in multiple dosing studies.^147^ A Swedish study also found a difference in the absorption *t*_1/2_ of tolterodine between EM (0.41 h) and PM (0.53 h), and EM were found to have a slight increase in heart rate at steady state in comparison with baseline, which was thought to be related to drug exposure.^148^ Interest in understanding drug‐induced QT interval prolongation led to study of the effect of CYP2D6 polymorphism on ECG changes in the use of tolterodine and its active metabolite 5‐HMT. In CYP2D6 PM, the systemic exposure to tolterodine was higher than in EM (*t*_1/2_ of tolterodine immediate release was 10 h in PM vs. 2 to 3 h in EM), which may have contributed to differences in ECG changes.^148^ However, the total concentration of active moieties (tolterodine plus 5‐HMT) was similar for PM and EM, which makes dose adjustment unhelpful for equalizing drug exposure. Interestingly, 5‐HMT and tolterodine may have contributed differently to QT interval prolongation risk and so this was studied as well. QT interval prolongation in CYP2D6 PM was only slightly greater for PM likely due to differences in protein binding between the two active components.^149^ As a further illustration of the impact of CYP2D6 genetic variation on anticholinergic pharmacokinetics, 4 mg daily dosing of fesoterodine produced a *C*_Max_ of 3.45 ng/ml in CYP2D6 PM versus 1.89 ng/ml in CYP2D6 EM. A similar proportional result was also observed for 8 mg daily dosing of fesoterodine in PM (*C*_Max_ of 6.40 ng/ml) versus EM (*C*_Max_ 3.98 ng/ml). Fesoterodine equally followed CYP2D6 and CYP3A4 metabolism which should lessen susceptibility to the effects of CYP2D6 reduced metabolism, but this was not been clearly demonstrated.^150^ The oral antimuscarinic agent darifenacin was metabolized by CYP3A4 and CYP2D6 with the main metabolite being inactive.^151^ The oral bioavailability of darifenacin was significantly altered by the CYP2D6 genotype in a dose‐dependent fashion. In EM, the bioavailability of 7.5, 15, and 30 mg CR oral doses of darifenacin were 15%, 19%, and 25%, respectively. In IM and PM, this bioavailability became 40%–90% higher. There was less impact of the CYP2D6 variants on the systemic elimination of darifenacin. In UM, the *t*_1/2_ of darifenacin was 3.12 h, while in PM it was 3.83 h.^119^

All told, CYP2D6 was an important contributor to variation in the pharmacokinetics of its substrates. In a study of patients with schizophrenia, Jürgens et al. reported that PM and UM did receive higher doses of medication, including CYP2D6‐dependent antipsychotics, than EM and IM. UM would likely need higher doses to compensate for their increased metabolism, so it was reassuring to see this in practice. However higher doses being used by PM may reflect adverse drug events being misinterpreted as psychotic symptoms leading to inappropriate and potentially harmful dose increases.^152^

#### 3.5.2 CYP2C19 and CYP3A4

Genetic polymorphisms in the CYP2C19 gene also result in PM, IM, and EM phenotypes. To date, no studies have demonstrated a role of CYP2C19 genetic variation in anticholinergic medication pharmacokinetics. Previous research has failed to identify individuals with no CYP3A4 activity. Due to the lack of genetic PM of CYP3A4, other factors such as exposure to drug inducers and inhibitors, liver function, blood flow, and possibly age and sex were the biggest considerations for variation in CYP3A4 activity.^66^, ^71^

## 4 DISCUSSION

Anticholinergic medications pose serious risks to older adults that include increased risk of cognitive impairment (including dementia). We know that adverse drug reactions are often proportional to plasma drug concentrations or for anticholinergic medications the total serum anticholinergic activity^14^, ^18^, ^153^ which makes the effects of sex, age, and CYP polymorphisms on drug disposition relevant for clinical decision‐making. While most of the studies described were small in size and short in duration, there are findings supportive that for certain anticholinergic medications in certain settings (most often increased age or female sex) there is a risk of increased anticholinergic medication exposure. Most notably that older adults experience increased exposure to bladder anticholinergics. Being aware of the potential for increased drug exposure and the potential associated risks should help clinical decision‐making regarding use of anticholinergic medications.

This review on the role of sex, age, and CYP polymorphism on anticholinergic medications confirmed that lower doses are preferable for some individuals. First, women often experience increased drug exposure^49^, ^122^ which likely contributes to their experience of more adverse drug reactions than men.^95^, ^97^, ^115^, ^116^, ^118^ Women can have other modifying factors such as increased age or CYP polymorphisms which can further potentiate their increased exposure to anticholinergic medications. While the tenants of Geriatric medicine have been relatively effective in communicating the importance of lower doses in older adults, the importance of sex in dosing has been poorly translated into clinical practice. Monographs frequently provide advice for dosing in the oldest users but rarely offer advice for dosing in women. Second, older age leads to alterations in drug metabolism and elimination that can also increase drug exposure. And third, clinical testing of CYP2D6 polymorphisms and adoption of peer‐reviewed published clinical practice guidelines for prescribing based on genotype^154^, ^155^, ^156^ where strong evidence exists may also help reduce the burden of adverse drug responses in older people. With increased risk of hospitalization, cognitive impairment, and mortality as risks from anticholinergic drug use, improved understanding of sex, age, and genomic testing of CYP isozymes may be indicated to reduce serious anticholinergic adverse events. Rigorous pharmacokinetic analysis is a much needed and important next step to allow us to understand how dosing recommendations can be modified to treat older men and women most safely and effectively. Studies done in the past often examined age, sex, or CYP polymorphisms alone and future work needs to account for all these factors in combination so that we may better approach personalized medicine for optimal outcomes.

## DISCLOSURE

There are no conflicts of interest to disclose for any author.

## AUTHOR CONTRIBUTION

Shanna Trenaman chose the topic of the review, developed the search strategy, completed the search, chose studies to include, drafted the document and finalized revisions, Kerry Goralski supported during the search and manuscript drafting and then revised the manuscript, Susan Bowles and Melissa Andrew revised the final draft supporting the clinical context in the review.

## Data Availability

Data sharing is not applicable to this article as no new data were created or analyzed in this study.

## 1

**TABLE A1:** Comprehensive table of anticholinergic drugs with pharmacokinetic considerations for age, sex, and CYP polymorphism

Generic drug name	ACB^1^	ARS^2^	ADME^3^	Effect of		
				Sex on ADME	Age on ADME	Genetics on ADME
Alprazolam	1		F: approximately 90% Distribution: 80%, mostly to albumin Metabolism: Liver, extensive via CYP3A Renal clearance: 371 ml/h Renal excretion: 80% Fecal excretion: 7% TBC: 76 ml/min *T*_1/2_: After oral administration to healthy adults 11.2 h	The weight‐normalized clearance of alprazolam is 20%–30% higher in young women than in young men	Renal clearance is significantly decreased in elderly men	
Amantadine	2	2	F: 86%–94% Distribution: 59%–67% bound to serum proteins *V*_d_: 404 L or 4.9 L/kg Metabolism: Liver, extensive via CYP3A Renal clearance: 371 ml/h Renal excretion: 80% Fecal excretion: 0.6% TBC: 0.2–0.3 L/h/kg *T*_1/2_: 17 (±4) h	Amantadine has significantly higher renal clearance in men	Reduced clearance in elderly patients and reduced renal function: 22.6–45 h	
Amitriptyline	3	3	F: high Metabolism: Liver, CYP1A2, CYP2C9, CYP2C19, CYP2D6, and CYP3A4 *T*_1/2_: 15 h (range: 9–25 h)	Amitriptyline plasma levels were higher in women than in men	1.5‐fold higher ratio of absolute serum concentration to dose adjusted serum concentration in the oldest age group in comparison to controls <40 years of age	
Atenolol	1		F: 46%–60% Distribution: <5% bound to serum proteins, brain tissue:blood concentration ratio of 0.2:1 *V*_d_: 50–75 L Metabolism: No liver metabolism and no active metabolites Renal excretion: 40%–50% Fecal excretion: 50% *T*_1/2_: 6–7 h			
Atropine	3	3	F: high Distribution: Serum protein binding is highly variable by age: 22.5% ±20.6% (<16 years), 14% ±9.1% (16–58 years), 22.2% ±16.7% (65–75 years) *V*_d_: 3.3–3.9 L/kg *T*_1/2_: 4 h (adults), 6.5 h (children)		Protein binding is highly variable upon age, *t*_1/2_ varies by age	
Baclofen		2	F: 100% *V*_d_: 59.1 L Metabolism: Liver, limited Renal clearance: 103 ml/min Renal excretion: 69%–85% of oral dose Fecal excretion: 10% TBC: 180 ml/min *T*_1/2_: 3–6.8 h			
Benztropine	3	3	F: poor			
Brompheniramine	3		*V*_d_: 11.7 L/kg Metabolism: Liver, extensive Renal excretion: 17% *T*_1/2_: 25 h			
Bupropion	1		Distribution: 84% bound to serum proteins, CSF concentration 10–25 fold higher than plasma *V*_d_: 19–21 L/kg Metabolism: Liver, extensive, primarily CYP2B6 Renal excretion: 87% Fecal excretion: 10% TBC: 160 ml/h (±23%) *T*_1/2_: 14–21 h	Mean AUC and *C*_Max_ for bupropion are higher in women than men however once these parameters are standardized for body weight the statistical significance is lost	In older adults (mean age 71.5 years) the clearance was 80% that seen in younger adults and the elimination *t*_1/2_ was extended to 34 h compared with most sources which report 11–14 h	
Captopril	1		F: 70%–75% Distribution: 25%–30% bound to serum proteins *V*_d_: 0.7 L/kg Metabolism: Liver, 50% Renal clearance: 0.4 L/kg/h Renal excretion: 95% TBC: 0.8 L/kg/h *T*_1/2_: 1.9 h			
Carbamazepine	2		F: 70%–79% Distribution: 76% bound to serum proteins, the CSF/serum ratio 0.22 *V*_d_: 0.8–2 L/kg Metabolism: Liver, 98%, extensive via CYP3A4, inducer of CYP3A4 and CYP1A2 Renal excretion: 72% Fecal excretion: 28% TBC: 80 ml/min *T*_1/2_: 12–17 h		Patients 70 years and older had a decreased clearance by approximately 70%	
Cetirizine	1	2	F: rapid and complete Distribution: 93% bound to serum proteins *V*_d_: 0.5–0.8 L/kg Metabolism: Liver, minimal Renal excretion: 60% Fecal excretion: 10% TBC: 53 ml/min *T*_1/2_: 7.4–9 h		The *t*_1/2_ is prolonged by 50% in older adults and in patients with chronic liver disease as compared with normal healthy adults	
Chlorpheniramine	3	3	F: good *V*_d_: 3.2 L/kg Metabolism: Liver, extensive Renal excretion: 50% Fecal excretion: <1% TBC: 234–470 ml/h/kg *T*_1/2_: 20 h			
Chlorpromazine	3	3	F: 32% Distribution: 90%–99% bound to serum proteins, CSF concentration 5 times the plasma concentration *V*_d_: 8–160 L/kg Metabolism: Liver, large extent Renal excretion: 23% *T*_1/2_: 6 h			
Cimetidine	1	2				
Clomipramine	3		F: 20%–78% Distribution: 97% bound to serum proteins, mostly albumin, CSF:plasma ratio is 2.6 *V*_d_: 7–20 L/kg Metabolism: Liver, extensive Renal excretion: 51%–60% Fecal excretion: 24%–32% TBC: 12.7–56.5 L/h *T*_1/2_: 19–37 h	The ratio of absolute serum concentration in comparison with the dose‐adjusted serum concentration is 1.1‐ to 1.5‐fold higher in women than in men, which suggests a dose reduction of 10%–30% for females	There is a 1.5‐fold higher ratio of absolute serum concentration to dose adjusted serum concentration in the oldest age group in comparison to controls <40 years of age	
Clozapine	3		F: 50%–60% Distribution: 97% bound to serum proteins *V*_d_: 6 L/kg Metabolism: Liver, extensive via CYP2D6, CYP1A2 and CYP3A4 Renal excretion: 50% Fecal excretion: 30% *T*_1/2_: 8–12 h	TBC differs between men and women: Men—36.7 L/h; Women—27 L/h	TBC differs by age at 39 years of age or older clearance is decreased by 0.219 L/h	
Codeine	1		Distribution: 7%–25% bound to serum proteins *V*_d_: 3–6 L/kg Metabolism: Liver, extensive by CYP2D6, CYP3A4 and UDP‐glucuronosyltransferases Renal excretion: 90% *T*_1/2_: 3 h			A specific CYP2D6 genotype are ultra‐rapid metabolizers (UM) of codeine who convert codeine into morphine, more rapidly and completely which may lead to higher than expected serum morphine levels, increasing the risk of overdose symptoms even at labeled doses
Colchicine	1		F: approximately 45% Distribution: 39% bound to albumin *V*_d_: 5–8 L/kg Metabolism: Liver, partial via CYP3A and p‐glycoprotein substrate Renal clearance: 0.727 L/h/kg Renal excretion: 40%–65% Fecal excretion: extensive TBC: 30.3 L/h *T*_1/2_: 26.6–31.2	In a single dose study, the plasma *t*_1/2_ in elderly males was 30 and 34 h in elderly females	Following a single oral dose of colchicine 0.6 mg, the mean apparent *t*_1/2_ was 24.92 ± 5.34 h for subjects age 18–30 years (*n* = 21) and 30.06 ± 10.78 h for subjects of mean age 62.83 years (*n* = 18)	
Cyclobenzaprine	2	2	F: 33%–55% Distribution: 93% bound to serum proteins Metabolism: Liver, extensive via P450 CYP3A4, CYP1A2, CYP2D6 Renal excretion: 51% TBC: 0.7 L/min *T*_1/2_: 18 h		In those >65 years of age receiving cyclobenzaprine hydrochloride extended release 30 mg capsules, the plasma *t*_1/2_ was prolonged (50 h) compared to younger subjects (32 h)	
Cyproheptadine	2	3	Metabolism: Liver 57% Renal excretion: 40% Fecal excretion: 2%–20% *T*_1/2_: 16 h			
Darifenacin	3		F: 15%–25% Distribution: 98% bound to serum proteins, mostly alpha‐1‐acid glycoprotein *V*_d_: 163 L Metabolism: Liver, extensive via CYP3A, CYP2D6 Renal excretion: 60% Fecal excretion: 40% TBC: 32–40 L/h *T*_1/2_: 13–19 h	Total body clearance is 31.1% lower in females than males		Approximately 7% of Caucasians and 2% of African Americans are poor metabolizers (PM) of CYP2D6 metabolized drugs which shunts its metabolism to CYP3A4, *C*_Max_/AUC for oral darifenacin 15 mg once daily at steady state was 1.9 for PM and 1.7 for extensive metabolizers (EM)
Desipramine	3	2	*V*_d_: 33–42 L/kg Metabolism: Liver, extensive Renal excretion: 70% *T*_1/2_: 14.3–24.7 h	Faster oral clearance in older men than older women	*T*_1/2_ is prolonged in older adults (*t*_1/2_ 30 h)	"Slow" metabolizers have a *t*_1/2_ 77 h
Desloratadine	1		Distribution: 82%–87% bound to serum proteins Metabolism: Liver, extensive via CYP2C8 Renal excretion: 40.6% Fecal excretion: 46.5% TBC: 150 L/h *T*_1/2_: 19–40 h			
Diazepam	1		F: ~98% Distribution: 95%–99.3% bound to serum proteins, CSF concentration is 1.6% of the total plasma concentration *V*_d_: 0.8–1 L/kg Metabolism: Liver, extensive Renal excretion:75% *T*_1/2_: up to 48 h	Protein binding is significantly greater in males than in females (1.87 L/kg in young females vs. 1.34 L/kg in young males) Greater clearance in women than men based on CYP3A4 clearance Shorter *t*_1/2_ in men compared to women (32 h vs. 46.2 h)	Protein binding is significantly greater in older females than younger females (2.46 L/kg in older females vs. 1.38 L/kg in younger females), the *V*_d_ is larger for older males than younger males (1.65 L/kg for older males vs. 1.19 L/kg for younger males), *t*_1/2_ increases by about 1 h for each year beginning with a *t*_1/2_ of 20 h at 20 years, the mean *t*_1/2_ increased with age to 79 h (range, 37–169 h)	
Dicyclomine	3	3	F: rapidly absorbed *V*_d_: 3.65 L/kg Metabolism: Liver, extensive via CYP3A Renal excretion: 79.5% Fecal excretion: 8.4% *T*_1/2_: 1.8 h			
Digoxin	1		F: 60%–80% Distribution: 25% bound to serum proteins, does cross the blood brain barrier *V*_d_: 475–500 L Metabolism: Liver 13%, substrate of p‐glycoprotein Renal excretion: 50%–70% Fecal excretion: 3%–5% *T*_1/2_: 36–48 h	Slower digoxin clearance in females	In the elderly, the *V*_d_ may be reduced, which could increase serum concentrations, elimination may occur more slowly in older adults, due to age‐related decline in renal function	
Dimenhydrinate	3		F: well absorbed Metabolism: Liver, extensive			
Diphenhydramine	3	3	F: 65%–100% Distribution: 76%–85% bound to serum proteins *V*_d_: 480–292 L/70 kg Metabolism: Liver 50% TBC: 11.7–49.2 ml/min/kg *T*_1/2_: 4–8 h			
Doxepin	3		Distribution: 80% bound to serum proteins *V*_d_: 11,930 L Metabolism: Liver, extensive via CYP2D6, CYP2C19 Renal Excretion: <3% *T*_1/2_: 15.3 h	Females had significantly higher dose‐corrected serum concentration doxepine/N‐doxepine (29%)	Patients older than 60 years had significantly higher dose corrected serum concentration of doxepin and N‐doxepin (48%), than patients up to 60 years	
Doxylamine	3		F: good *T*_1/2_: 10.1–13.11 h		*T*_1/2_ in older men (mean age, 66 years) is 15.5 ± 2.1 h, in older women (mean age, 73 years), the *t*_1/2_ was longer than in young women, but the difference was not statistically significant (12.2 h vs. 10.1 h)	
Fentanyl	1		Distribution: 80%–86% bound to serum proteins *V*_d_: 4–6 L/kg Renal excretion: <7% Fecal excretion: 1%–9% TBC: 42–53 L/h *T*_1/2_: 3–27 h (depending on dosage form)			
Fesoterodine	3		F: 52% Distribution: 50% bound to serum proteins *V*_d_: 169 L Metabolism: Liver, extensive via CYP2D6, CYP3A Renal excretion: 70% Fecal excretion: 7%		In older adults, renal clearance of fesoterodine is reduced	
Fluvoxamine	1		F: 53% Distribution: 80% bound to serum proteins, mostly albumin *V*_d_: 25 L/kg Metabolism: Liver, extensive, Inhibitor of CYP1A2, CYP2C9, CYP2C19, CYP2D6, and CYP3A4 Renal excretion: 94% Fecal excretion: 7% *T*_1/2_: 15.6–16.3 h	Higher serum concentration in women than men at 100 mg orally	In older patients the clearance of fluvoxamine was reduced by 50%	
Furosemide	1		F: 47%–70% Distribution: 91%–99% bound to serum proteins, mostly albumin *V*_d_: 0.2 L/kg Metabolism: Liver 10% Renal clearance: 2 ml/min/kg Renal excretion: 60%–90% Fecal excretion: 7%–9% TBC: 76 ml/min *T*_1/2_: 30–120 min		*T*_1/2_ is prolonged in older adults	
Haloperidol	1	1	F: 60%–70% Distribution: >90% bound to serum proteins *V*_d_: 9.5–21.7 L/kg Metabolism: Liver, extensive via CYP3A Renal excretion: 33%–40% Fecal excretion: 15%			
Hydralazine	1		F: 38%–50% Distribution: 88%–90% bound to serum proteins *V*_d_: 0.3–8.2 L/kg Metabolism: Liver, extensive Renal excretion: 3%–14% Fecal excretion: 3–12% *T*_1/2_: 3–5 h			
Hydrocortisone	1		F: 96% Distribution: 90% bound to serum proteins, mostly corticosteroid‐binding globuli *V*_d_: 34 L Metabolism: Liver, extensive via CYP3A Renal excretion: extensive TBC: 18 L/h *T*_1/2_: 1–2 h			
Hydroxyzine	3	3	*V*_d_: 16 L/kg Metabolism: Liver *T*_1/2_: 3–20 h		A mean *t*_1/2_ of 29.3 h was reported after administration of 0.7 mg/kg hydroxyzine syrup to 9 healthy, fasting adults mean age 69.5 years	
Hyoscyamine	3	3	F: complete Renal excretion: majority unchanged *T*_1/2_: 7.47 h			
Imipramine	3	3	F: 94%–96% Distribution: 89% bound to serum proteins *V*_d_: 10–20 L/kg Metabolism: Liver, extensive via CYP2C19 *T*_1/2_: 6–18 h		In older adults *t*_1/2_ ranges from 25–30 h	
Isosorbide	1		F: approximately 100% Distribution: <5%% bound to serum proteins *V*_d_: 0.6–0.7 L/kg Metabolism: Liver 98% Renal clearance: 371 ml/h Renal excretion: 93% Fecal excretion: 1% TBC: 115–140 ml/min *T*_1/2_: 5 h			
Loperamide	1	2	F: 0.3% Renal excretion: 1% Fecal excretion: 25%–40% *T*_1/2_: 7–15 h			
Loratadine	1	2	Distribution: 97% bound to serum proteins Metabolism: Liver, extensive via CYP3A, CYP2D6 *T*_1/2_: 12–15 h		Older adults (*n* = 12) reported to have a *t*_1/2_ of 17.5 h (range of 11–38 h)	
Loxapine	2		F: complete Distribution: 96.6% bound to serum proteins Metabolism: Liver, extensive via CYP1A2, CYP3A4, CYP 2D6, p‐glycoprotein inhibitor *T*_1/2_: 17.6 h			
Meperidine	2		Distribution: 65%–80% bound to serum proteins, mostly albumin and alpha‐1‐acid glycoprotein *V*_d_: 3.1–5 L/kg Metabolism: Liver, extensive *T*_1/2_: 3.2–3.7 h		In older adults, meperidine is less protein bound; however, the clearance rate is unchanged, therefore the *V*_d_ may be greater with more available free drug, and in older adults the *t*_1/2_ is extended	
Methocarbamol	3	1	F: completely Metabolism: Liver, extensive Renal excretion: 10%–15% Fecal excretion: small amount *T*_1/2_: 0.9–2 h			
Metho‐trimeprazine	2		*V*_d_: 29.8 L/kg Metabolism: Liver Fecal excretion: small amount *T*_1/2_: 15 h			
Metoprolol	1		F: 50% Distribution: 10% bound to serum albumin, CSF concentration close to the plasma concentration *V*_d_: 3.2–5.6 L/kg Metabolism: Liver, extensive via CYP2D6 Renal excretion: 95% *T*_1/2_: 3–4 h			In CYP2D6 PM the mean *t*_1/2_ of metoprolol is 7–9 h
Morphine	1		F: 20%–40% Distribution: 20%–36% bound to serum proteins *V*_d_: 1–6 L/kg Metabolism: Liver Renal excretion: 90% Fecal excretion: 7–10% TBC: 20–30 ml/min/kg *T*_1/2_: 1.5–4.5 h			
Nifedipine	1		F: complete Distribution: 92%–98% bound to serum proteins Metabolism: Liver, extensive via CYP3A4 Renal excretion: 80% Fecal excretion: 20% TBC: 4.3 ml/min/kg *T*_1/2_: 2 h	Greater clearance in women, mainly due to CYP3A4 and is 20%–30% higher in young women than young men, women reach higher plasma levels at same dose	Clearance is significantly reduced in older subjects (unrelated to renal function) compared to younger subjects, following IV administration clearance in older subjects was 348 ml/min compared with 519 ml/min in young subjects	
Nortriptyline	3	2	F: 60% Distribution: 86%–95% bound to serum proteins *V*_d_: 15–27 L/kg Metabolism: Liver, extensive via CYP2D6 Renal excretion: 2% *T*_1/2_: 15–39 h	Plasma levels are mostly affected by CYP2D6 genotype and sex with females experiencing higher plasma levels	The *t*_1/2_ may be >90 h in older adults	Nortriptyline plasma levels are mostly affected by CYP2D6 genotype and sex with females experiencing higher plasma levels
Olanzapine	3	2	F: well absorbed Distribution: 93% bound to serum proteins, mostly albumin and alpha‐1‐acid glycoprotein *V*_d_: 1,000 L Metabolism: Liver, extensive via CYP1A2, CYP2D6 Renal excretion: 57% Fecal excretion: 30% TBC: 26.1 L/h *T*_1/2_: 21–54 h	Men from a population including individuals with Alzheimer's disease or schizophrenia cleared olanzapine 38% faster than women	The mean *t*_1/2_ was 1.5 times greater in healthy patients aged ≥65 years compared with younger patients age <65 years, according to a study of 24 healthy subjects	
Orphenadrine	3		F: 95% Renal excretion: 60% *T*_1/2_: 13.2–20.1 h			
Oxcarbazepine	2		Distribution: 40% bound to serum proteins *V*_d_: 49 L Metabolism: Liver, extensive Renal excretion: >95% Fecal excretion: <4% *T*_1/2_: 2 h			
Oxybutynin	3	3	F: 6% Distribution: >99% bound to serum proteins, mostly alpha‐1‐acid glycoprotein Metabolism: Liver, extensive via CYP3A4 Renal excretion: <0.1% *T*_1/2_: 2–3 h	Oxybutynin was not shown to have any differences in AUC and *C*_Max_ for men or women	Oxybutynin follows the trend of increasing peak plasma levels and bioavailability with increasing age and frailty	
Paroxetine	3	1	F: complete Distribution: 93%–95% bound to serum proteins Metabolism: Liver, extensive via CYP2D6, also an inhibitor of CYP2D6 Renal excretion: 64% Fecal excretion: 36% TBC: 76 ml/min *T*_1/2_: 15–21 h	Sex is correlated to paroxetine plasma concentration, estimates of *V*_2_ in male subjects were 461.30 ± 259.75 and in female subjects were 346.41 ± 255.81	A naturalized study of paroxetine showed a 2‐fold higher ratio of absolute serum concentration to dose adjusted serum concentrations in the oldest age group in comparison to controls <40 years of age	
Perphenazine	3	3	F: 20% *V*_d_: 10–34 L/kg Metabolism: Liver, extensive via CYP2D6 Renal excretion: 80% TBC: 100 L/h *T*_1/2_: 8.4–12.3 h			
Prednisone	1		F: 92% Distribution: 70% bound to serum proteins, mostly albumin and corticosteroid‐binding globuli *V*_d_: 0.4–1 L/kg Metabolism: Liver, extensive *T*_1/2_: 2–3 h			
Quetiapine	3	1	F: 100% Distribution: 83% bound to serum proteins *V*_d_: 10 L/kg Metabolism: Liver, extensive via CYP3A4 Renal excretion: 73% Fecal excretion: 20% *T*_1/2_: 6–7 h	Sex was not shown to effect pharmacokinetics of quetiapine	In a pharmacokinetic study, quetiapine clearance was reduced by 40% in patients ≥65 years (*n* = 9) compared with young patients (*n* = 12)	
Quinidine	1		F: 70%–80% Distribution: 80%–88% bound to serum proteins, mostly albumin and alpha‐1‐acid glycoprotein *V*_d_: 2–3 L/kg Metabolism: Liver, extensive via CYP3A4 Renal clearance: 1 ml/min/kg Renal excretion: 5%–20% Fecal excretion: 1%–3% TBC: 3–5 ml/min/kg *T*_1/2_: 6–8 h	Women clear quinidine at a faster rate than men and women have ECG changes in response to drug activity much quicker than men which is not explained by quinidine clearance		
Ranitidine	1	1	F: 50% Distribution: 15% bound to serum proteins *V*_d_: 1.04–4.09 L/kg Metabolism: Liver, minor Renal clearance: 24.6–31.8 L/h Renal excretion: 3%–70% Fecal excretion: 3.1 ml/min/kg TBC: 1.29–1.44 L/h/kg *T*_1/2_: 1.9–3 h		The *t*_1/2_ is 3–4 h in older adults after oral administration likely due to a decrease in renal function	
Risperidone	1	1	F: 70% Distribution: 90% bound to serum proteins *V*_d_: 1–2 L/kg Metabolism: Liver, extensive via CYP 2D6 Renal clearance: 0.96 L/h Renal excretion: 70% Fecal excretion: 14% TBC: 3.2–13.7 L/h *T*_1/2_: 3–20 h	Sex‐related differences in risperidone metabolism are unlikely to be significant	When the plasma concentration was adjusted for subject body weight or maintenance dose there were still significant differences between groups with the oldest group having the highest adjusted concentration	Polymorphisms of CYP2D6 are more responsible for variation in risperidone metabolism than sex
Scopolamine	3		Metabolism: extensive Renal excretion: <10% *T*_1/2_: 9.5 h			
Solifenacin	3		F: approximately 90% Distribution: 98% bound to plasma proteins, primarily alpha‐1‐acid glycoprotein *V*_d_: 599–671 L Metabolism: Liver, extensively via CYP3A4 Renal clearance: 0.67–0.76 L/h Renal excretion: 3%–6% Fecal excretion: 22.5% TBC: 9.4 L/h *T*_1/2_: 40–68 h		Solifenacin has a longer *t*_1/2_ due to slower elimination and to longer time to reach *C*_Max_ in older adults, this can be explained by the reduced absorption of solifenacin in older adults. Exposure to solifenacin is increased about 1.2‐fold in older subjects	
Theophylline	1		F: well absorbed Distribution: 40% bound to serum proteins *V*_d_: 450 ml/kg Metabolism: Liver, extensive via CYP1A2 Renal excretion: 10%–13% Fecal excretion: 7% TBC: 76 ml/min *T*_1/2_: 8.7 h		Protein binding is reduced in older adults, older adults had reduced clearance 0.59 ± 0.07 ml/kg/min, and increased mean *t*_1/2_ of 9.8 h (1.6–18 h) this was in healthy older non‐smokers and was not significantly different from clearance values in otherwise healthy non‐smoking younger asthmatics	
Thioridazine	3	3	*V*_d_: 17.8 L/kg Metabolism: Liver, extensive Renal excretion: small amounts *T*_1/2_: 21–24 h			
Tolterodine	3	2	F: 77% *V*_d_: 113 L Metabolism: Liver, extensive via CYP2D6 Renal excretion: 77% Fecal excretion: 17% *T*_1/2_: 1.9–3.7 h			Metabolism is slowed in individuals who are CYP2D6 PM as metabolism is shunted to CYP3A4, *t*_1/2_ is prolonged to 6.5 h with single doses and 9.6 h with multiple doses
Trazodone	1	1	F: 65% Distribution: 89%–95% bound to serum proteins *V*_d_: 0.47–0.84 L/kg Metabolism: Liver, extensive Renal clearance: 3–5.3 L/h Renal excretion: 70%–75% Fecal excretion: 21% TBC: 5.3 L/h *T*_1/2_: 7 h			
Triamterene	1		F: 30%–70% Distribution: 55%–67% bound to serum proteins Metabolism: Liver 80% Renal excretion: 21% *T*_1/2_: 1.5–2.5 h		*T*_1/2_ was 4.3 h in young adults, and was prolonged to 6.5 h in an older patient	
Trifluoperazine	3	3	F: readily absorbed Distribution: 90%–99% bound to serum proteins Metabolism: Liver *T*_1/2_: 24 h			
Trospium	3		F: 9.6% Distribution: 50%–85% bound to serum proteins *V*_d_: 395 L Metabolism: Liver Renal clearance: 29.07 L/h Renal excretion: 5.8% Fecal excretion: 85.2% *T*_1/2_: 20 h			
Venlafaxine	1		Distribution: 27%–30% bound to serum proteins *V*_d_: 7.5 L/kg Metabolism: Liver, extensive via CYP2D6 Renal clearance: 0.074–0.079 L/h/kg Renal excretion: 87% Fecal excretion: 2% TBC: 1.3 L/h/kg *T*_1/2_: 5 h	Venlafaxine serum concentrations differed in men and women with higher concentrations achieved by women (215 and 151 nmol/L), the ratio of absolute serum concentration in comparison to the dose‐adjusted serum concentration is 1‐ to 1.5‐fold higher in women than in men	The concentration to dose ratio of venlafaxine was 1.5‐fold higher in adults over 65 in comparison with controls <40 years old	The serum concentration of N‐desmethyl‐venlafaxine was 5.5‐fold higher in a subset of CYP2D6 PMs (*p* <.01) and 22‐fold higher in second subset of CYP2D6 PMs (*p* <.001) than in EM
Warfarin	1		F: completely absorbed Distribution: 99% bound to serum proteins *V*_d_: 0.14 L/kg Metabolism: Liver, extensive via CYP2C9, CYP2C19, CYP2C8, CYP2C18, CYP1A2, CYP3A4 Renal excretion: 92% TBC: dependent on CYP2C19 genotype *T*_1/2_: 1 week			

Abbreviations: ACB, Anticholinergic Cognitive Burden Scale; ADME, Absorption, Distribution, Metabolism and Excretion; ARS, Anticholinergic Risk Scale; AUC, area under the curve; CYP, cytochrome P450; F, Bioavailability; IV, intravenous; TBC, Total Body Clearance.
